# Optimized DINO model for accurate object detection of sesame seedlings and weeds

**DOI:** 10.1038/s41598-025-96826-6

**Published:** 2025-04-07

**Authors:** Yong Wang, ShunFa Xu, ZhenYuan Ye, KongHao Cheng

**Affiliations:** Computer Science and Technology, Zhejiang University of Technology, Hangzhou, 310000 China

**Keywords:** Object detection, DINO, Multi-scale features, Attention mechanism, Machine learning, Field trials

## Abstract

The application of intelligent agricultural machinery is crucial in modern agricultural production. However, in environments where the target and the surrounding morphology are highly similar, such as distinguishing sesame seedlings from weeds, the problem essentially becomes one of optimizing edge detection algorithms for similar targets. To address this issue in agricultural object detection, we developed a custom dataset containing 1,300 images of sesame seedlings and weeds. To overcome the high complexity and low detection accuracy limitations of the original DINO model for this problem, the backbone network was replaced with MobileNet V3, the SENet attention mechanism and neck structure were optimized, and the H-Swish6 activation function was introduced to suit edge devices. Given the higher degree of lignification in the stems of sesame seedlings, these modifications improved the overall Average Precision (AP) of the model on the COCO dataset by 5.1% compared to the original DINO model. Specifically, $\text {AP}_{S}$ and $\text {AP}_{M}$ increased by 3.3% and 3.8%, respectively, while $\text {AP}_{50}$ and $\text {AP}_{75}$ increased by 2.3% and 3.2%. The model’s parameter count was reduced to 29M, inference time was lowered by 60%, and computational cost in FLOPs decreased by 43.72%. To verify the effectiveness of the improvements, we developed a custom dataset containing 1,300 images of sesame seedlings and weeds. On this model, the improved DINO model achieved a maximum AP of 81.8%, outperforming the YOLOv7 model by 5.6%, with an FPS of 24 frames per second. Ablation experiments verified the effectiveness of the model improvements.However, the aforementioned studies have not addressed the issue of low detection accuracy in scenarios with similar targets in the agricultural domain.

## Introduction

According to The State of Food and Agriculture 2023^1^,the hidden costs of agriculture, including weeds, account for approximately 10% of global GDP. With the rapid advancement of modern agricultural technology, accurately distinguishing between crops and weeds in environments where the target and surrounding morphology are highly similar has become increasingly important, playing a crucial role in agricultural automation. From the Transformer-based DETR (Detection with Transformers) architecture to its derivatives and further improved object detection models^2^, DINO (DETR with Improved deNoising anchOr boxes) undeniably stands out as a leader in this field^3^. This area has attracted extensive scholarly research, leading to the development of numerous innovative and impactful methodologies. According to the relevant literature, these modeling approaches can accurately identify crops and weeds, thereby enhancing crop yields while reducing the use of chemical herbicides, ultimately mitigating adverse ecological impacts^4–6^. However, despite the abundance of research, significant challenges in practical agricultural environments remain inadequately resolved: these models exhibit suboptimal performance when processing real-world data on agricultural edge devices.

In agricultural automation technology, accurately distinguishing between crops and weeds in real-world environments remains an urgent issue to address^7^. Although existing methods theoretically demonstrate excellent performance, in practical applications, captured images often present a high degree of similarity in appearance and pixel size between sesame seedlings and weeds, creating unique classification challenges. This visual proximity results in low recognition rates for traditional object detection models when differentiating between the two. The problem is especially pronounced in the early growth stages of sesame seedlings, where their similarity to weeds is even more evident, further complicating classification^8^. These factors collectively limit the effectiveness of these models in practical agricultural applications, such as pesticide spraying or weed removal robots. In recent years, deep learning models have been increasingly applied in agricultural tasks, such as plant classification and disease detection. For instance, Gulzar et al.^9^demonstrated robust performance in soybean classification by modifying the Inception model through the integration of transfer learning, adaptive learning rate adjustment, and model checkpointing techniques. Similarly, Amriet al.^10^ created a new dataset and proposed the MIV-PlantNet model, achieving a precision of 96% and an F1 score of 98%, highlighting its robustness and reliability, and advancing the development of automated plant classification systems in Saudi Arabia. Alkanan^11^ proposed an enhanced iteration of the MobileNetV2 model, strategically incorporating additional layers and applying model optimization techniques, achieving an accuracy of approximately 96%, outperforming state-of-the-art (SOTA) models in precision, recall, and F1 score.

Furthermore, Gulzar et al.^12^provide a comprehensive understanding of the trends and advancements in the field of fruit classification using deep learning, which informs future research endeavors. Gulzar et al.^13^ proposed a novel deep learning-based method for seed classification. The experimental results showed that the pre-trained Xception model using transfer learning performed exceptionally well, achieving a perfect classification accuracy of 1.0000 on both the validation and test sets, with significantly reduced training loss and faster convergence. This achievement provides an efficient technical pathway for automated seed quality detection and crop breeding optimization. Additionally, Seelwal^14^ offered insights into significant research efforts in rice disease detection over the past decade, emphasizing the importance of evaluation accuracy and advocating for the implementation of hybrid deep learning and machine learning methods to enhance disease recognition. This aligns with our approach of utilizing deep learning for crop and weed detection in complex agricultural environments. .

Zhu et al.^15^ addressed the challenges of dense scenes by introducing a Transformer-based prediction head to YOLOv5, enhancing its detection capabilities across different scales. Wang’s team combined YOLOv5’s grid search technique with YOLOX’s^16^ decoupled head design, resulting in the more performant YOLOv7 algorithm^17^. Within DETR-like algorithms, DDOD and DDQ, proposed by Chen^18^ and Zhang^19^ respectively, further explored the issues of decoupling and query selection in object detection, achieving significant progress in small object detection and crowded scenes as well as in the one-to-one assignment strategy for dense queries. Zhang et al.^20^ introduced the Curriculum Pseudo Label Transformer method, which utilized Data Augmentation Consistency and pseudo labels to effectively enhance DINO’s adaptive object detection capabilities across multiple domains, demonstrating its strong potential in handling complex visual data.Suneel et al.^21^ proposed a detection system based on deep neural network (DNN) to process weed images. The detected weeds would be located and removed by an equipped weeder. The system was evaluated on the maize field image dataset, and the results showed that it could detect and remove weeds with an accuracy of more than 80%.

This paper focuses on the effective classification of sesame seedlings and weeds, a task that inherently addresses the scientific problem of improving object detection accuracy. Given the practical application needs of this technology-especially in the field of intelligent agricultural machinery-clear requirements for lightweight models have been established. Therefore, this paper proposes an improved sesame seedling and weed detection method based on the modified DINO model, with an emphasis on enhancing detection accuracy.

First, the DINO model was adjusted by replacing the backbone network to reduce the number of neural network layers. Second, the attention mechanism was improved, and the activation function was redesigned, followed by the redesign of multi-scale feature extraction. These modifications collectively improved the model’s accuracy and efficiency. The model effectively provides precise classification decisions for pesticide spraying and weed removal robots in real agricultural environments, enhancing operational efficiency and accuracy. Therefore, in view of the above problems and based on the above research results, this article proposes an improved DINO model for sesame seedling and weed detection in agricultural scenarios. The main contributions are as follows:

1. The original ResNet backbone network is replaced with MobileNet V3 to reduce the number of parameters and computational costs while maintaining detection accuracy.
2. The SENet attention mechanism is enhanced to prioritize channels with higher weights during feature extraction.
3. A novel H-Swish6 activation function is designed to accelerate model convergence, thereby improving the adaptability and overall performance of the model on edge devices.

## Methods

### Experimental data

The experiments in this paper used the COCO dataset and a custom sesame seedling and weed dataset.

#### COCO dataset

The COCO (Common Objects in Context) dataset is a dataset used for computer vision research^22^. It encompasses 91 object categories, 328,000 images, and 2.5 million labels. This is particularly important for the training of object detection algorithms. Moreover, the COCO dataset is widely used in the training and evaluation of deep learning models, especially in the field of object detection. Many influential papers and algorithms that have been published use the COCO dataset for validation, making it an important standard for measuring model performance. Validating algorithms on the COCO dataset provides a better understanding of the model’s performance in real scenarios, facilitating its improvement and optimization.

#### Self-made sesame seedling and weed dataset

This thesis employs a self-made dataset of sesame and weed images. A total of 589 field photos of sesame seedlings and weeds were captured using a Canon 5D Mark III camera. The location is the Sesame Estate in Changshu Village, Anyang Township, Chun’an County, Hangzhou City. The shooting time was in September 2023. All the related sesame and weed images presented in this thesis are from this dataset. The photos were collected in clear weather conditions, using a standard 50mm lens at approximately 0.5 meters distance. During the course of the research, we did not harm these plants in any way, only photographing them for documentation purposes. The original photos were taken as 4000x3000 pixel color images. After collection, photos that adversely affected the detection model were removed, leaving 546 images. The original size of the images was too large for the training of object detection models, so the images were converted to a size of 512x512.

In the Sesame Seedling and Weed Dataset, we present a comparison of the bounding box counts for two different categories: Sesame-crop and Weed. The total number of bounding boxes for each category is shown in Table 1. Specifically, the number of bounding boxes for Weed is 430, while the number for Sesame-crop is 606.

**Table 1:** Bounding box counts for Sesame-crop and Weed.

Category	Total Bounding Boxes
Sesame-crop	606
Weed	430

**Fig. 1:**
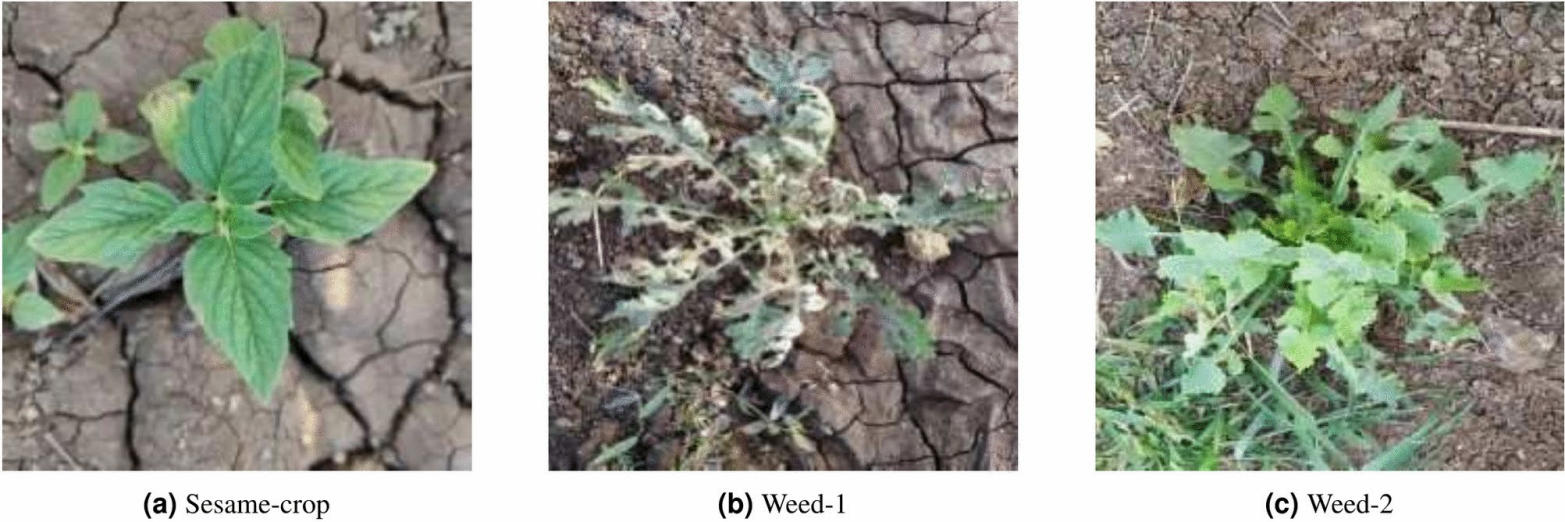
Custom dataset images.

Then, image preprocessing was carried out on the 546 original images using the ImageDataGenerator data augmentation technique in the Keras neural network framework, expanding the number to 1,300 images, with Figure 1 showing a selection of images from the self-constructed Sesame Weed Dataset. The image preprocessing involved several data augmentation techniques to enhance the robustness and diversity of the training dataset:

Rotation: Images were randomly rotated within a range of ±30 degrees to simulate varying orientations of the sesame seedlings and weeds. Horizontal Flip: Images were randomly flipped horizontally to increase the model’s robustness to left-right symmetrical changes. Zoom: Images were randomly scaled between 0.8 and 1.2 times to mimic changes in shooting distance. Width and Height Shift: Images were randomly translated within ±20% of the image dimensions to enhance the model’s adaptability to positional changes. Shear: Images were randomly sheared within a range of ±20 degrees to simulate changes in shooting angles.

The sesame seedlings and weeds dataset was manually annotated using Labelimg annotation software, with bounding boxes for weeds and sesame seedlings. The generated annotation information files were of txt type, and finally, they were converted into COCO-type annotation information files through automated scripts.

To validate the robustness and generalization of the model in specific environments of sesame seedlings and weeds, the experiments were repeated 5 times. Additionally, we employed 5-fold cross-validation (k=5) to ensure comprehensive evaluation. In this approach, the dataset was divided into five subsets, and the model was trained and validated five times, each time using a different subset as the validation set and the remaining four subsets as the training set. The results from these multiple experiments were averaged, and standard deviations were calculated to provide a more reliable assessment of model performance.

### Research methods

#### DINO network

From the Transformer-based DETR to its derivatives and further refined object detection models, DINO is the latest iteration. Specifically, DINO is a DETR-like model that introduces a new framework through improvements in denoising training, query initialization, and bounding box prediction. As a DETR-like structure, DINO consists of a backbone network^23^, a multi-layer Transformer encoder, a multi-layer Transformer decoder, and multiple prediction heads. Unlike traditional DETR, DINO uses dynamic anchor boxes as queries in the decoder, gradually refining these anchors across multiple decoder layers. Additionally, to enhance stability during training, DINO incorporates ground truth labels alongside noisy bounding boxes into the Transformer decoder layers to optimize the bipartite matching process. DINO also introduces deformable attention mechanisms to improve computational efficiency and accuracy when processing irregularly shaped objects. These innovations enable DINO to improve detection accuracy while optimizing training and inference efficiency compared to previous models.

**Fig. 2:**
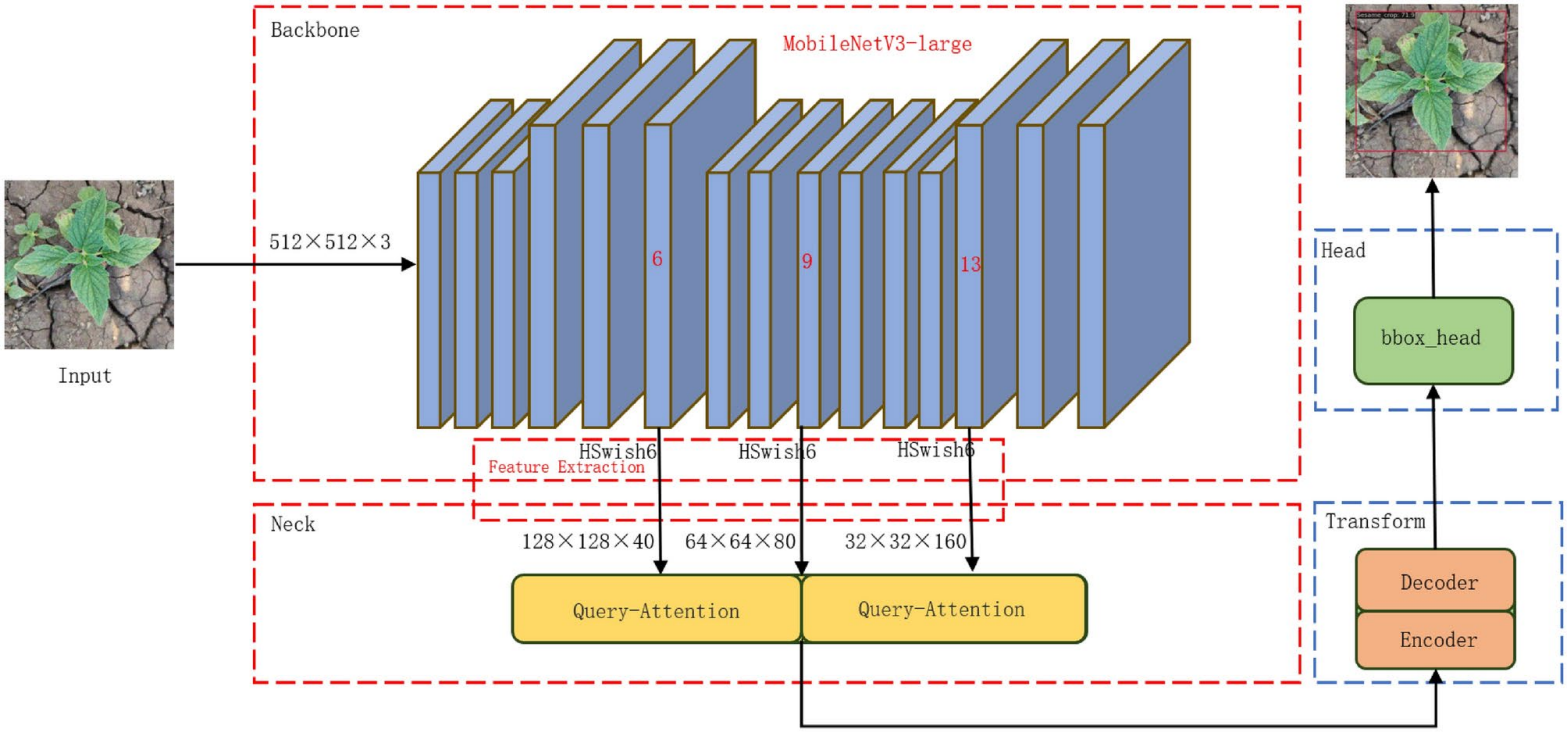
Schematic diagram of improved DINO network structure.

Despite these advantages, the original DINO design employs a ResNet backbone network, which, due to its fixed architecture, limits the model’s size flexibility and lacks specialized feature extraction training for specific scales^24^. This results in slow inference speed and high background false detection rates in similarly sized object detection tasks, such as sesame seedling and weed detection. Therefore, this study introduces a series of improvements to the DINO model, including replacing the backbone network to accommodate different image processing needs and optimizing the attention mechanism to enhance feature extraction accuracy and efficiency. Moreover, intensive ablation studies were conducted in the model’s Neck section to explore more effective feature fusion methods and improve the model’s ability to identify small-scale targets. The improved overall architecture is shown in Figure 2(The sesame seedling image shown in the figure is from the self-constructed sesame weed dataset).

#### Backbone network design

Compared to heavyweight neural networks with high complexity and many parameters, mobile networks are better suited for storage- and power-constrained applications such as edge devices due to their smaller parameter count, lower computational requirements, and faster inference speed. With technological advancements and growing societal demands, the use of edge devices is expanding, particularly in the agricultural object detection field, making the pursuit of mobile networks crucial. This enables embedding more convenient and efficient object detection algorithms into edge devices to enhance on-site computation efficiency and operability.

In this context, DINO, an improved DETR-like network in the one-stage detection series, introduces major innovations in training methods and algorithm updates. This study aims to improve the DINO model, making it more suitable for agricultural applications, particularly for detecting sesame seedlings and weeds on edge devices, addressing real-world limitations and challenges. Therefore, DINO was chosen as the primary research framework, with necessary module improvements and algorithm optimizations applied to propose a model specifically tailored for sesame seedling and weed detection. This model not only meets the resource constraints of edge devices but also optimizes detection efficiency and accuracy.

MobileNet V3, the latest iteration of the classic mobile convolutional neural network series, builds on the results of the V1 and V2 versions and further improves the basic building blocks. It integrates the SE module to leverage channel attention mechanisms for enhanced recognition and capture of key features. Furthermore, MobileNet V3 employs a redesigned H-Swish activation function that simplifies the Swish function to reduce computational complexity and improve derivative efficiency, thereby enhancing the model’s operational efficiency. The tail structure of the model has also been optimized to reduce redundant computation. Compared to similar mobile networks like GhostNet, MobileNet V3 employs a Neural Architecture Search (NAS) strategy optimized for hardware performance, enabling automatic network structure adjustments for specific platforms, ensuring optimal performance on various agricultural mobile terminals and meeting the adaptive needs of agricultural applications.

**Fig. 3:**
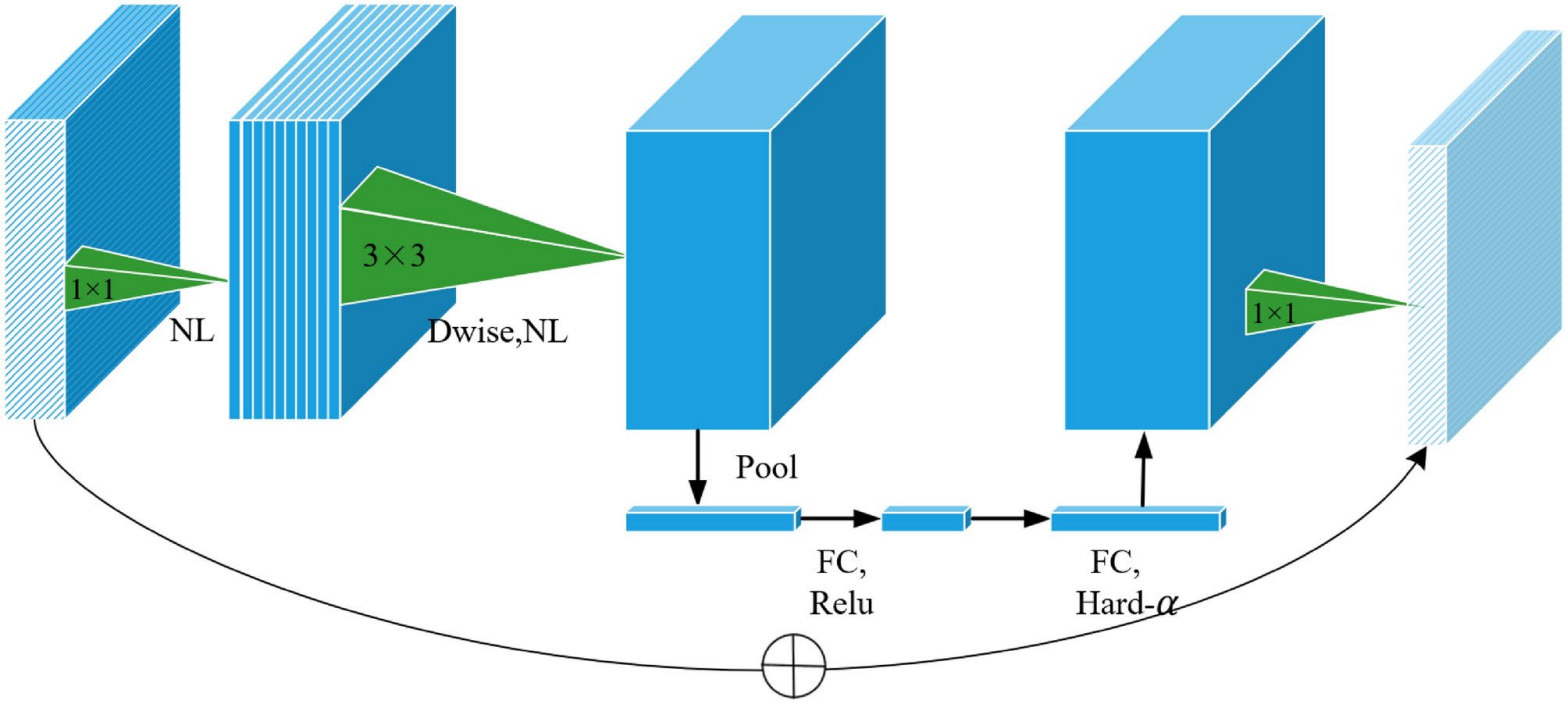
Schematic diagram of MobileNet V3 core modules.

Thus, this paper replaces DINO’s original backbone network ResNet with MobileNet V3, reducing the network layers by 70% to further decrease computational load and improve speed. MobileNet V3’s core modules primarily implement depthwise separable convolution kernels, SE channel attention mechanisms, and residual connections, as illustrated in Figure 3.

After analyzing the MobileNet V3 network structure and conducting extensive experiments, the final improvement plan included two main components: First, max pooling was incorporated into the attention mechanism to enhance the capture of key features. The improved mechanism divides input into global and average pooling branches to avoid losing crucial features in a single pooling process, thereby retaining more relevant information. Second, the original H-Swish activation function was optimized to address potential instability during gradient updates and to adapt to precision-constrained devices, ultimately accelerating the training process.

#### Attention mechanism improvement

In terms of the attention mechanism, the original MobileNet V3^25^ used the SENet channel attention mechanism to measure the importance of each channel in the feature map. By using global average pooling, the model extracted global information from the feature map, aiming to reduce redundancy and enhance the model’s generalization capability. This study proposes adding a global max pooling branch to enhance the model’s sensitivity to key image regions and reduce interference from irrelevant information, thereby improving the ability to localize target areas.

**Fig. 4:**
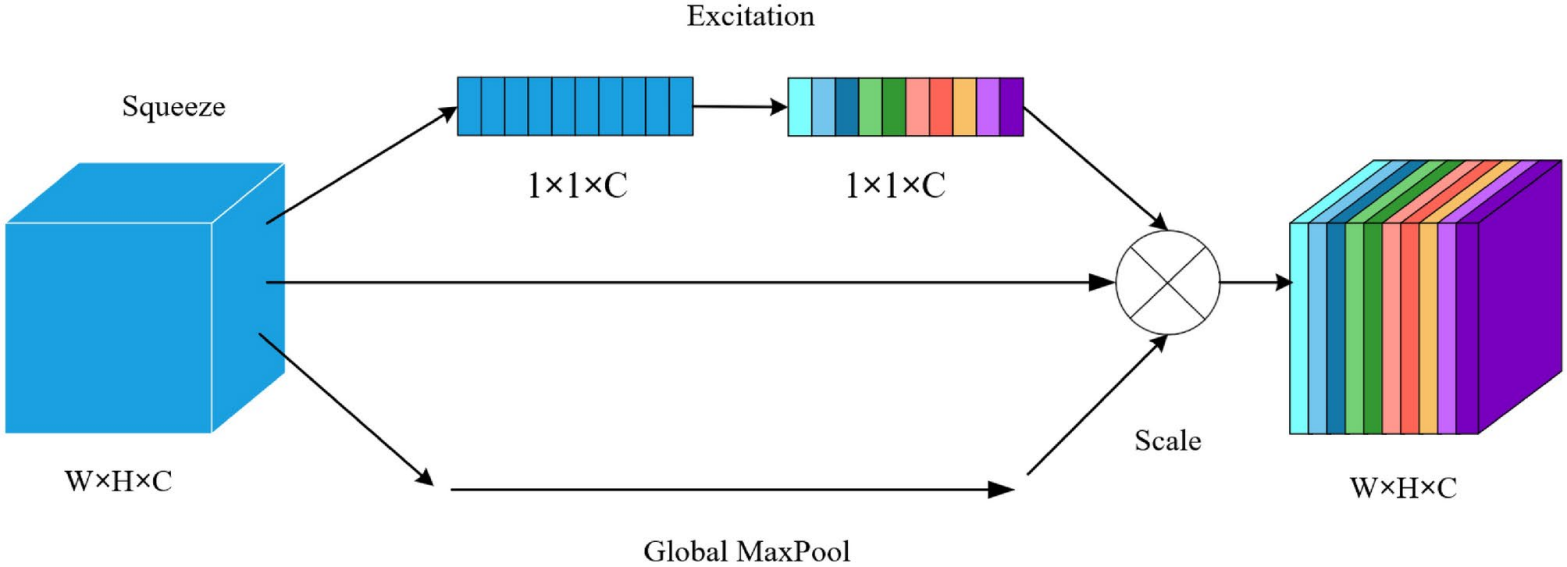
Schematic diagram of improved SENet structure.

Thus, the main improvement to SENet is the introduction of a global max pooling branch. This enhancement helps capture salient features in the image more effectively while preserving texture information. By merging the outputs of the new branch, the model can capture a broader range of image features. Then, two fully connected layers learn the weights of different features, improving the accuracy of the DINO-based object detection model. The optimized SENet structure proposed in this paper is shown in Figure 4:

As shown above, before introducing the SE attention mechanism, the importance of each channel in the feature map was assumed to be equal. By integrating the SE module, the neural network can differentiate the importance of different channels and assign them varying weights, enabling the model to prioritize channels with greater weight during feature extraction. The improvements made in this study take into account the diversity of target scales and features in agricultural settings, especially for detecting sesame seedlings and weeds.

Global Average Pooling (GAP) is a dimensionality reduction operation applied to the final layer of a convolutional neural network. It calculates the average value of all pixels in the feature map, converting a 2D feature map into a single value. The mathematical expression is:

$$\begin{aligned} GAP = \frac{1}{H \times W} \sum _{i=1}^{H} \sum _{j=1}^{W} F_{ij} \end{aligned}$$

where H is the height of the feature map, W is the width, and ${F_{ij}}$ is the value of the pixel at (i, j). GAP retains global information while reducing the number of parameters to avoid overfitting. Although global average pooling can integrate global feature information from the image, it may also introduce interference from a significant amount of irrelevant background information.

Global Max Pooling (GMP), on the other hand, selects the maximum value from the feature map as output. Its mathematical expression is:

$$\begin{aligned} GMP = {\max _{i,j}}{F_{ij}} \end{aligned}$$

The advantage of GMP lies in highlighting the most salient features of the target object and being more robust to background noise and interference. Comparison between the two: GAP preserves global information but reduces attention to the target; GMP focuses on the strongest features of the target but may lose details. In our study, GAP introduces a large amount of agricultural background noise (e.g., soil, dead leaves, and cotton fibers), while GMP effectively highlights the key features of sesame seedlings and suppresses background interference. Therefore, we chose GMP.

**Fig. 5:**
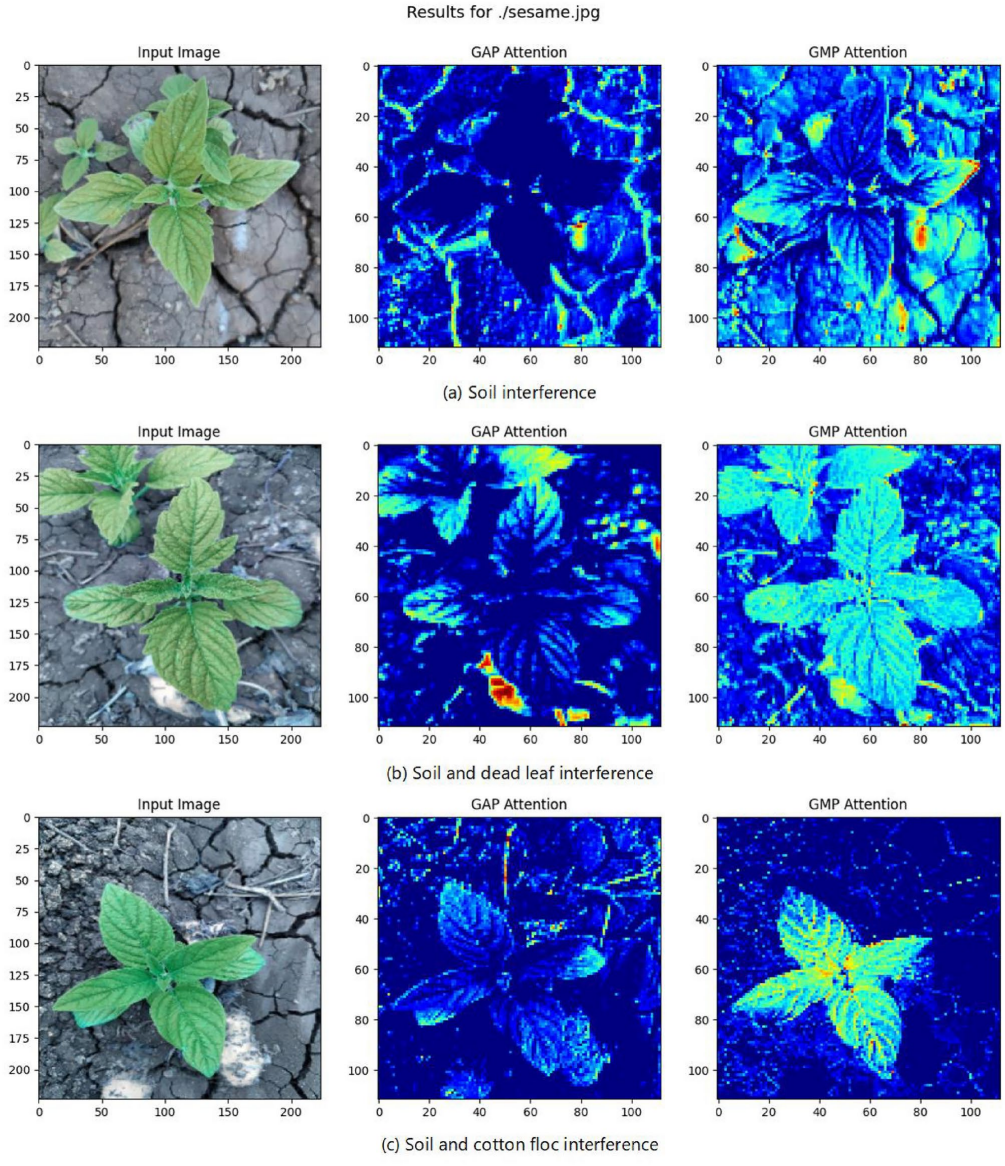
Grad-CAM Comparison of Sesame Seedlings under Global Max Pooling and Global Average Pooling.

Grad-CAM (Gradient-weighted Class Activation Mapping) is a technique for visualizing the regions of interest in a neural network. It generates a heat map based on gradient information to show which regions of the input image the model focuses on during prediction. Through Grad-CAM visualization experiments, we systematically compared the attention distribution between GAP and GMP in the sesame seedling recognition task (as illustrated in Figure 5, the sesame seedling image presented is sourced from our self-constructed Sesame Weed Dataset):

Figure 5(a): In GAP, the sesame seedling region appears darker, while the background is brighter, indicating interference from soil. GMP significantly enhances the model’s attention to the stems and leaves of the sesame seedlings.

Figure 5(b): In GAP, the high-intensity red areas focus on dead leaves. GMP reduces attention to dead leaves and enhances focus on sesame seedlings.

Figure 5(c): GMP makes the sesame seedling region much brighter than GAP, while effectively suppressing attention to background soil and cotton fibers.

The experimental results fully demonstrate that GMP significantly enhances the model’s attention to key morphological features of sesame seedlings while effectively suppressing interference from the complex farmland background. This is fully consistent with our biological hypothesis for improving the attention mechanism.

Given that sesame plants are Pedaliaceae with more upright stems than typical weeds, and that slightly mature seedlings possess lignified stems, this paper incorporates global max pooling to emphasize the salient features of sesame seedlings while reducing interference from irrelevant noise and details. This enhancement improves the model’s performance in identifying agricultural targets.

Additionally, this paper considered that embedding the attention mechanism module directly into the backbone network might interfere with already optimized weights, affecting feature extraction efficiency and target detection accuracy. Therefore, the improved attention mechanism module was placed at the output of critical feature layers in the backbone network to maintain stable weight values and improve detection accuracy. This strategy aims to balance the model’s global perception ability and precise extraction of target-specific features, enhancing the model’s capability for recognizing and classifying agricultural targets without compromising the backbone network’s core weights.

#### Activation function design

In terms of activation function design, the original MobileNet V3 used the H-Swish activation function. Considering the visual similarity between sesame seedlings and weeds, and aiming to reduce computational costs on edge devices, speed up training convergence, and prevent overfitting, this paper proposes a new activation function, H-Swish6.

In the context of sesame seedling and weed detection, H-Swish6 offers significant advantages over the original H-Swish. While H-Swish simplifies the Swish function using a piecewise linear approximation, it lacks an upper bound, which can lead to unbounded activation values. Unrestricted activation values typically result in slow convergence, particularly when dealing with extreme feature values, which can cause accuracy loss, especially on resource-constrained devices.

In contrast, H-Swish6 introduces an upper bound to prevent the activation values from growing indefinitely. This modification ensures a more stable learning process for the network and accelerates convergence, especially in regions of the feature space dealing with extreme values. As a result, H-Swish6 reduces the risk of slow convergence and improves the overall performance of the model. This is particularly important for object detection in datasets such as sesame seedlings and weeds, where small or overlapping features are often difficult to detect.

**Fig. 6:**
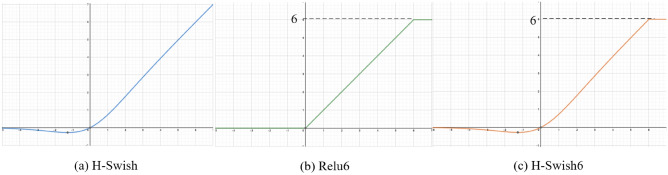
Schematic diagram of activation function curve.

Moreover, the upper bound control in H-Swish6 enables the model to learn more complex features without sacrificing computational efficiency. This allows it to perform better on tasks like sesame seedling and weed detection, especially on edge devices, which typically have limited processing power while requiring real-time performance and accuracy.The activation function curve is shown in Figure 6.

Through the aforementioned backbone network replacement, attention mechanism improvement, and activation function modification, this study optimized the DINO model for sesame seedling images. These adjustments made the model more suitable for handling agricultural images with highly similar features. Subsequent experimental results verified the effectiveness of the improvements, enhancing accuracy in sesame seedling detection tasks and demonstrating the importance of carefully adjusting existing deep learning architectures to fit specific application scenarios.

#### Multi-scale feature extraction design

After optimizing the DINO model’s backbone network with improved attention mechanisms and activation functions, this study continued to explore model components tailored for sesame seedling images. The backbone network is responsible for extracting basic feature information, while the Neck structure further refines these features. To meet the requirements of multi-scale object detection, an improved feature pyramid structure was designed, integrating feature layers of different scales within the backbone network to facilitate the fusion of multi-level features, effectively combining local and global features and enhancing multi-scale contextual feature representation.

**Fig. 7:**
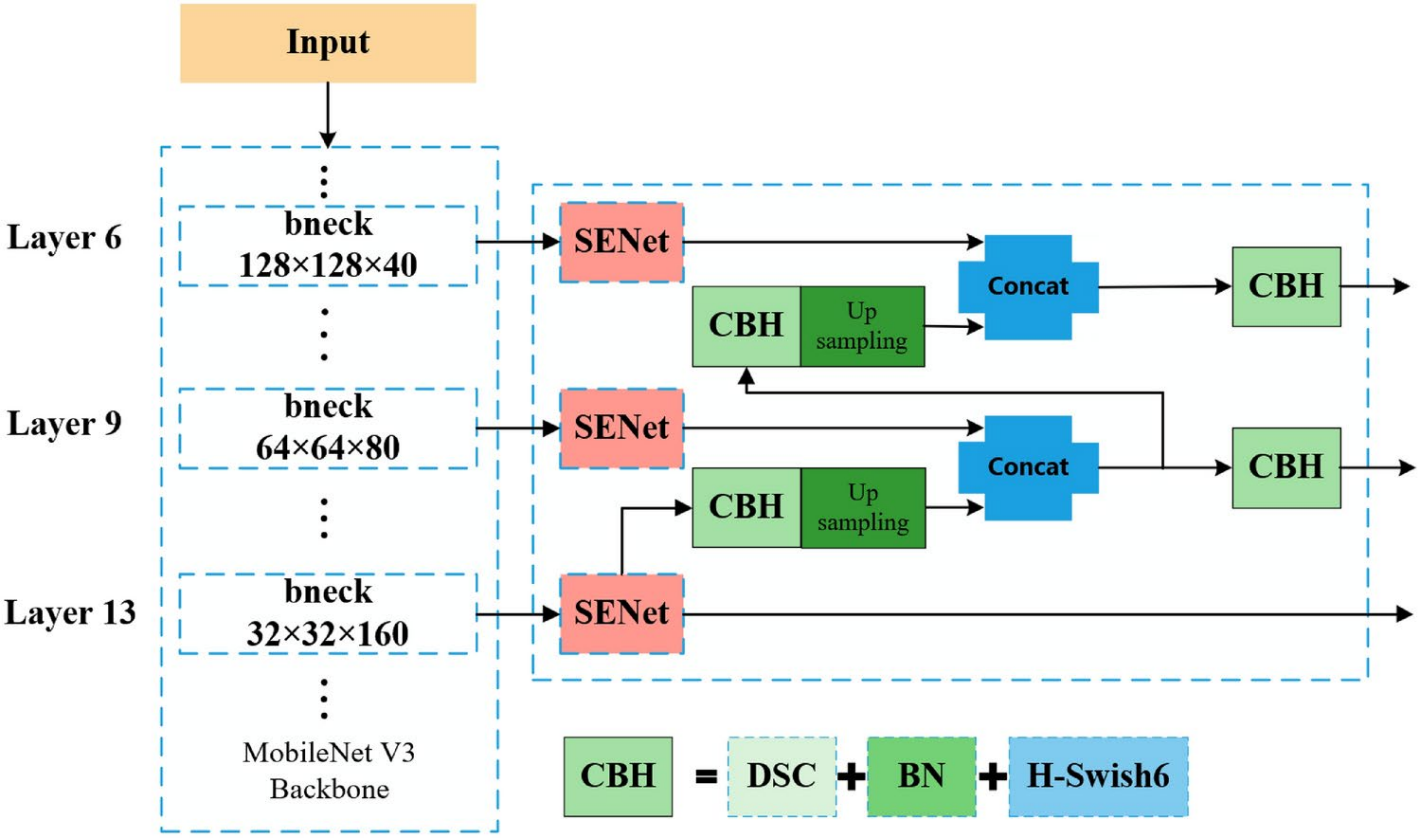
Improved multi-scale feature extraction structure diagram.

Given that the original DINO model used ResNet as the backbone network and the previous section replaced it with MobileNet V3 to reduce network complexity and improve mobile device compatibility, the multi-scale feature extraction strategy was accordingly adjusted. To address the limitations of computing resources on edge devices, the number of feature extraction layers was reduced from four specific levels in ResNet to three levels in MobileNet V3 (layers 6, 9, and 13) to simplify the model and reduce the computational burden.

Inspired by the FPN (Feature Pyramid Network) + PAN (Path Aggregation Network) structure in the YOLOv5 model^26^, the improvement plan also includes adding upsampling modules and extra 3$\times$3 convolution layers to mitigate potential adverse effects from downsampling fusion. Compared to the original DINO structure, this improvement plan aims to enhance the detection accuracy of sesame seedling targets in the agricultural field while reducing the overall model parameters. The final improvement plan and its effect are shown in Figure 7.

In the newly replaced DINO backbone network, feature extraction is performed at layers 6, 9, and 13. The activation function in the Convolution-Batch Normalization-Activation (CBH) structure was replaced with the H-Swish6 function designed in this study. Furthermore, the Deep Separable Convolution (DSC) module was used for depthwise separable convolution to optimize the network structure and improve computational efficiency.

## Results

###  Results for the improved DINO network model

To comprehensively evaluate the overall performance of the improved DINO network model, joint training and validation were performed on both the COCO dataset and a custom sesame seedling and weed dataset. As shown in Table 2, this study trained and tested seven network models on the COCO dataset, including DETR, Deformable DETR, DAB-DETR, Dynamic DETR, DN-DETR, the original DINO, and the improved Our-Model^27–30^. These seven network models were evaluated based on target detection rates, where $\text {AP}_{S}$, $\text {AP}_{M}$, and $\text {AP}_{L}$ represent the average precision for objects smaller than 32$\times$32 pixels, between 32$\times$32 and 96$\times$96 pixels, and larger than 96$\times$96 pixels, respectively.

Before conducting this comparison, convergence evaluations were performed on all models involved. During the experiments, it was observed that the DETR model converged after 227 epochs, while Deformable DETR, DAB-DETR, Dynamic DETR, and DN-DETR^31^ showed signs of convergence after 103, 101, 97, and 121 epochs, respectively. This determination was based on the stability of the loss function values and performance metrics. The original DINO and the improved Our-Model demonstrated higher training efficiency, showing signs of convergence after only 47 epochs.

The decision to compare the models after 47 epochs was made to highlight the improved Our-Model’s efficiency and superiority over shorter training periods. This choice not only illustrates the convergence speed of each model but also ensures fairness and scientific rigor in the comparison.

**Table 2:** Performance metrics of DETR-like models on the COCO.

Model	Epoch	mAP	$\text {mAP}_{50}$	$\text {mAP}_{75}$	$\text {mAP}_{S}$	$\text {mAP}_{M}$	$\text {mAP}_{L}$
DETR	227	18.5	32.4	17.5	7.3	18.1	29.7
Deformable DETR	103	44.1	-	-	-	-	-
DAB-DETR	101	41.0	63.3	42.8	22.2	43.9	58.4
Dynamic DETR	97	45.9	64.0	49.3	27.6	47.9	57.4
DN-DETR	121	46.4	64.9	50.2	27.8	49.8	62.4
DINO	47	52.0	69.6	56.5	35.0	55.3	66.0
Our-Model	47	**57.1**(+5.1)	**71.9**(+2.3)	**59.7**(+3.2)	**38.3**(+3.3)	**59.1**(+3.8)	**68.8**(+2.8)

The data in the table clearly show that the improved Our-Model demonstrates significant improvements across multiple performance evaluation metrics. As a crucial measure of object detection performance, the overall Average Precision (AP) of the improved Our-Model is 5.1% higher than the original DINO model. Specifically, $\text {AP}_{S}$ and $\text {AP}_{M}$, representing small and medium-scale object detection, improved by 3.3% and 3.8%, respectively, highlighting the model’s comprehensive enhancement in detecting targets of various scales. Notably, performance increases under different IoU thresholds (0.5 and 0.75), represented by $\text {AP}_{50}$ and $\text {AP}_{75}$, improved by 2.3% and 3.2%, respectively. This further validates the model’s significant advantage in accurately identifying and locating targets with different levels of overlap. These results demonstrate the significant improvements in accuracy and efficiency of the Our-Model compared to the original DINO model, particularly in scenarios involving moderately and highly overlapping objects.

In the comparison presented in Table 3, replacing the model’s backbone network with the more lightweight MobileNet V3 significantly optimized the overall structure. In terms of parameters measured in millions (M), this adjustment reduced the parameter count from 47M to 29M while markedly improving computational efficiency compared to the original DINO model. While maintaining high performance, the model’s inference time was reduced from 100 milliseconds in the original DINO to 40 milliseconds, a 60% decrease. Additionally, the computational cost in FLOPs (floating-point operations) was reduced to 112G, a reduction of 87G or about 43.72%, enhancing computational efficiency. Thus, the model achieved higher computational efficiency and faster inference speeds without compromising performance. It’s worth noting that DETR and DAB-DETR employ the DC5 (dilated convolutions) structure, while Deformable DETR, Dynamic DETR, DN-DETR, and DINO uniformly adopt the 4-scale multi-scale strategy.

**Table 3:** Complexity metrics of DETR-like models on the COCO.

Metric/Model	DETR	Deformable DETR	DAB-DETR	Dynamic DETR	DN-DETR	DINO	Our-Model
Inference Time	50 ms	42 ms	58 ms	61 ms	47 ms	100 ms	40 ms
FLOPS	195G	166G	256G	240G	183G	199G	112G
Params	41M	40M	44M	58M	48M	47M	29M

In this study, a performance comparison analysis was conducted on several widely used object detection models based on a custom sesame seedling and weed dataset to simulate challenges commonly encountered in real-world environments. As shown in Table 4, the different training epochs highlight the time variation required for each model to reach convergence. Convergence speed is a crucial indicator of training efficiency, influenced by factors such as model architecture, optimization algorithm, and dataset characteristics. The improved Our-Model in this study displayed a distinctive convergence pattern: it was slower than traditional Faster R-CNN and Mask R-CNN models but faster than the latest YOLOv5 and YOLOv7 models. Several factors can explain this phenomenon. First, Faster R-CNN and Mask R-CNN are region proposal-based networks, enabling them to quickly locate regions of interest during the early training stages, thereby accelerating convergence. However, this approach may face performance bottlenecks when processing large-scale datasets.

The model proposed in this paper employs a Transformer-based architecture that emphasizes global contextual information integration, providing an advantage in extracting complex features and processing large-scale datasets. While this approach initially increases convergence time, it ultimately bolsters detection accuracy. Despite YOLOv5 and YOLOv7’s reputation for rapid detection, these models require more time to adjust weights when handling datasets with complex backgrounds and similar objects, particularly when dealing with new and improved dataset structures. Thus, the improved DINO model achieves convergence speeds between those of traditional Faster R-CNN and Mask R-CNN models and the advanced YOLO series, balancing high-precision detection with training efficiency.

This balance highlights the importance of selecting an appropriate model based on specific practical requirements, and the improved DINO model offers an effective solution for balancing accuracy and speed. Regarding performance metrics, the selected models showed clear differences: the widely used Faster R-CNN and Mask R-CNN models underperformed on this dataset, with AP values of only 59.6% and 65.9%, respectively. Conversely, the YOLO series, particularly YOLOv5 and YOLOv7, exhibited superior accuracy, with YOLOv7 achieving an AP value of 76.2%.

Notably, the Our-Model proposed in this paper achieved exceptional performance on this dataset, with an AP value of 81.8%, surpassing YOLOv7 by 5.6 percentage points while also reaching a frame rate (FPS) of 24 frames per second. This result indicates that Our-Model not only surpasses existing technology in accuracy but also provides a faster response speed for real-time detection applications. This superior performance is attributed to the design features of the Our-Model, including optimizations for practical application scenarios and efficient multi-scale object processing capabilities. This demonstrates the significant advancements and practical value of this research in object detection technology.

**Table 4:** Performance Comparison Between Our-Model and General Models on mAP and FPS.

Model	Epoch	mAP	FPS
Faster-RCNN	37	59.6	21
Mask R-CNN	33	65.9	18
YOLOv5	65	74.3	23
YOLOv7	61	76.2	22
DINO	47	75.8	21
Our-Model	**47**	**81.8(+5.6)**	**24**

**Fig. 8:**
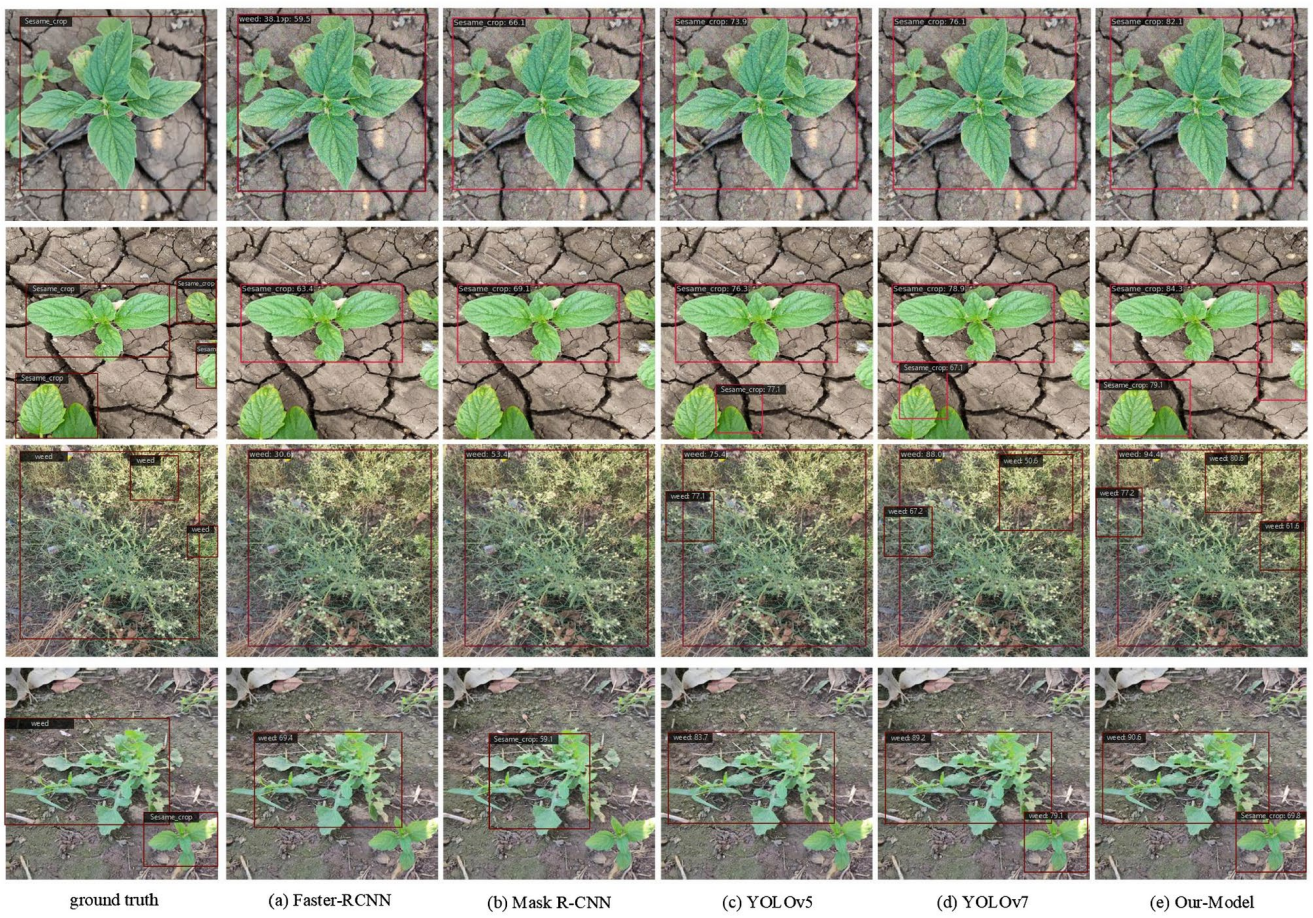
Visualization of sesame seedling and weed detection results.

To visually demonstrate the effectiveness of the improved DINO model in detecting sesame seedlings and weeds in complex and dense agricultural scenes, this paper conducted a series of visual comparative analyses, comparing it to current leading object detection models such as Faster R-CNN, Mask R-CNN, YOLOv5, and YOLOv7^32–35^. As shown in Figure 8(The sesame seedling images and weed images shown in the figure are all from the self- constructed sesame weed dataset), the detection results of Faster R-CNN, Mask R-CNN, YOLOv5, YOLOv7, and the improved Our-Model are sequentially presented.

In the first column, the ground truth of sesame seedlings and weeds is shown, while the last few columns represent the results of different models recognizing the images. In the first row, which features a single sesame seedling, the improved DINO model achieved an 82.1% recognition accuracy, significantly outperforming Faster R-CNN, which occasionally misclassified sesame seedlings and weeds as the same category. Compared to the YOLO series, this model improved recognition accuracy by at least 6%. In the second row, depicting a complex scene with multiple sesame seedlings, Faster R-CNN and Mask R-CNN often failed to identify all seedlings, mostly recognizing individuals with intact shapes. While the YOLO series could perform multi-object detection, it struggled to detect sesame seedlings with significant occlusion. In contrast, the improved DINO model not only performed effective multi-object detection but also accurately identified and marked occluded sesame seedlings.In the third row, a weed-dense scene, the model demonstrated similar excellent performance in detecting multiple weeds as it did with sesame seedlings, with a recognition rate significantly higher than other models and an accurate determination of weed distribution. In the fourth row, depicting a scene with both sesame seedlings and weeds, Mask R-CNN misidentified weeds with similar morphology as sesame seedlings, and YOLOv7 mistakenly recognized the sesame seedling in the lower right corner as a weed. However, the improved DINO model successfully distinguished between the sesame seedlings and weeds, achieving a weed recognition accuracy of 90.6% and a sesame seedling recognition accuracy of 69.8%.

###  Ablation experiment

To more accurately evaluate the impact of the improved SENet channel attention mechanism and multi-scale feature extraction Neck structure, this paper employed three comparison strategies: I: Remove both the SENet and Neck improvements. II: Retain the SENet improvements while removing the Neck improvements. III: Remove the SENet improvements while keeping the Neck improvements.

These experiments focused on comparing performance differences before and after the module improvements. By using MobileNet V3 as the backbone network and replacing only the SENet and Neck structures proposed in this paper while keeping other components consistent with the original model, the effects of these modifications could be highlighted more effectively. This approach ensured a direct comparison of the improvements, providing clearer insights into the contributions of each modification to the model’s performance. The results of these ablation studies on the sesame seedling and weed dataset are shown in Table 5.

According to the table, adopting the Neck structure designed in this paper reduced the overall parameter count (Params) by approximately 15.2% while maintaining the original model’s performance and increasing the mean Average Precision (mAP) by 5.5%. Although the improved SENet structure increased the parameter count by about 2.2%, it also resulted in a 1.7% improvement in mAP compared to the original model. Combining these two improvements, the overall parameter count was reduced by 13.0% relative to the original model, while accuracy achieved a 6% overall increase.

**Table 5:** Comparison results of SENet and Neck structure ablation experiments.

Backbone Network	Model	Improved SENet	Improved Neck	Params	mAP
Improved MobileNet V3	I	–	–	5.51M	81.4
	II	$\checkmark$	–	5.63M	83.7
	III	–	$\checkmark$	4.67M	86.9
	Our-Model	$\checkmark$	$\checkmark$	5.32M	87.4

## Discussion

To address the issue of low accuracy when deploying deep neural network image recognition algorithms on agricultural intelligent machinery edge devices, specifically in environments where sesame crops and their surroundings are similar, this paper proposes a sesame seedling and weed detection model based on DINO. The model incorporates MobileNet V3 modules and an improved multi-scale feature extraction Neck network to validate the effectiveness of these modifications. Since sesame seedlings have more upright stems and a degree of lignified stalks compared to common weeds, the SENet attention mechanism was optimized and combined with global max pooling to highlight the features of sesame seedlings. Additionally, to accommodate the needs of edge devices, this study introduces a new activation function, H-Swish6, and uses the PAFPN structure in the Neck network to reduce the computational load, alleviating potential issues arising from downsampling fusion by adding downsampling modules and 3$\times$3 convolutions. By carefully selecting specific layers, the multi-scale feature extraction strategy was optimized to match the image characteristics of sesame seedlings.

The experimental results show that these improvements increased the overall Average Precision (AP) on the COCO dataset by 5.1% compared to the original DINO model, with $\text {AP}_{S}$ and $\text {AP}_{M}$ increasing by 3.3% and 3.8%, respectively, and $\text {AP}_{50}$ and $\text {AP}_{75}$ improving by 2.3% and 3.2%, respectively. The model’s parameter count was reduced to 29M, inference time was cut by 60%, and the computational cost in GFLOPs decreased by 43.72%. On the custom sesame seedling and weed dataset, the model achieved a maximum AP of 81.8%, outperforming the YOLOv7 model by 5.6% and reaching an FPS of 24 frames per second. Ablation studies confirmed the effectiveness of the model improvements.

## Data Availability

The datasets generated and/or analyzed during the current study are not publicly available due to the subsequent need for the sesame crop dataset to serve as the data set for a graduation thesis. However, these data are available from the corresponding author upon reasonable request.
